# Identification of a novel DNMT1 mutation in a Chinese patient with hereditary sensory and autonomic neuropathy type IE

**DOI:** 10.1186/s12883-018-1177-2

**Published:** 2018-10-20

**Authors:** Wenxia Zheng, Zhenxing Yan, Rongni He, Yaowei Huang, Aiqun Lin, Wei Huang, Yuying Su, Shaoyuan Li, Victor Wei Zhang, Huifang Xie

**Affiliations:** 1 Department of Neurology, Zhujiang Hospital, Southern Medical University, 253 Industrial Avenue Middle, Guangzhou City, 510282 Guangdong Province China; 2 Department of Neurology, Nanfang Hospital, Southern Medical University, 1838 Guangzhou Avenue North, Guangzhou, Guangzhou, 510515 China; 3 AmCare Genomics Lab, Guangzhou, Guangzhou, 510320 China; 4 Department of Molecular and Human Genetics, Baylor College of Medicine, Houston, TX 77030 USA

**Keywords:** DNMT1, HSAN1E, Exome sequencing

## Abstract

**Background:**

DNA methyltransferase 1 (EC 2.1.1.37), encoded by DNMT1 gene, is one of key enzymes in maintaining DNA methylation patterns of the human genome. It plays a crucial role in embryonic development, imprinting and genome stability, cell differentiation. The dysfunction of this group of enzymes can lead to a variety of human genetic disorders. Until now, mutations in DNMT1 have been found to be associated with two distinct phenotypes. Mutations in exon 20 of this gene leads to hereditary sensory and autonomic neuropathy type IE, and mutations in exon 21 cause autosomal dominant cerebellar ataxia, deafness and narcolepsy.

**Case presentation:**

Here we report a novel DNMT1 mutation in a sporadic case of a Chinese patient with cerebellar ataxia, multiple motor and sensory neuropathy, hearing loss and psychiatric manifestations. Furthermore, we elucidated its pathogenic effect through molecular genetics studies and revealed that this defective DNMT1 function is responsible for the phenotypes in this individual.

**Conclusion:**

Our findings expand the spectrum of DNMT1-related disorders and provide a good example of precision medicine through the combination of exome sequencing and clinical testing.

**Electronic supplementary material:**

The online version of this article (10.1186/s12883-018-1177-2) contains supplementary material, which is available to authorized users.

## Background

DNA methyltransferase 1 (EC 2.1.1.37), encoded by DNMT1 gene, is one of key enzymes in maintaining DNA methylation patterns of the human genome. It plays a crucial role in embryonic development, imprinting and genome stability, cell differentiation [1, 2]. The dysfunction of this group of enzymes can lead to a variety of human genetic disorders. Human DNMT1 consists of a large N-terminal region harboring multiple conserved domains and a conserved C-terminal catalytic core. The large N-terminal region is composed of the DMAP1-binding domain (DNA methyltransferase-associated protein 1), the proliferating cell nuclear antigen-binding domain (PBD), the replication focus targeting sequence (TS) 4 domains, the CXXC domain, and two bromo-adjacent homology (BAH) domains [3]. Until now, all the mutations are found to be located in TS. TS is dispensable for the selective DNA methylation activity, but it can keep DNA from gaining access to the catalytic center and undergoing methylation by inserting into the catalytic pocket stably [4, 5]. So far mutations in DNMT1 have been reported to cause two distinct neurological syndromes: hereditary sensory and autonomic neuropathy type IE with dementia and deafness (HSAN1E) related to mutations in exon 20 and autosomal dominant cerebellar ataxia, deafness and narcolepsy (ADCA-DN) with hearing loss and narcolepsy related to mutations in exon 21 [1].

## Case presentation

A 38-year-old female from Guangdong province in China was admitted to Zhujiang Hospital, Southern Medical University in June 2016. At the age of 30, she developed progressive poor gait balance so that she frequently fell down when walking. At that time, she went to a local hospital for treatment, but diagnosis was not established. She was transferred to the Second Hospital Affiliated to Guangzhou Medical Hospital for hospitalization on December in 2010, where she was clinically diagnosed as cerebellar atrophy, Type 2 Diabetes and hyperlipemia. However, the treatments prescribed did not prevent disease worsening. In 2015, she presented a slowly progressing retardation. Within the year 2016, she began to suffer from bad-response, psychiatric manifestations, bilateral hearing loss and intermittent convulsion in her upper limb during sleeping, especially the right upper limb. With such complex symptoms, she was referred to our hospital. Her parents were not consanguinity, and no neurological disorders were found in her family members except herself.

Physical examination showed that she had mild mental retardation, apathy and spoke few words. Cranial nerves were normal except for symmetric bilateral sensory hearing loss. She did not cooperate with the neurological examination and sensibility could not be tested. Her muscle strength of bilateral upper limbs was normal but was decreased in lower limbs. Muscle tone was normal, but her right upper limb had abnormal involuntary movement. The patient had no pyramidal signs. Cerebellar function examination showed mild abnormalities on finger- to- nose, heel- to-knee and rapidly alternating pronation and supination of hands. Romberg test was negative.

**Fig. 1:**
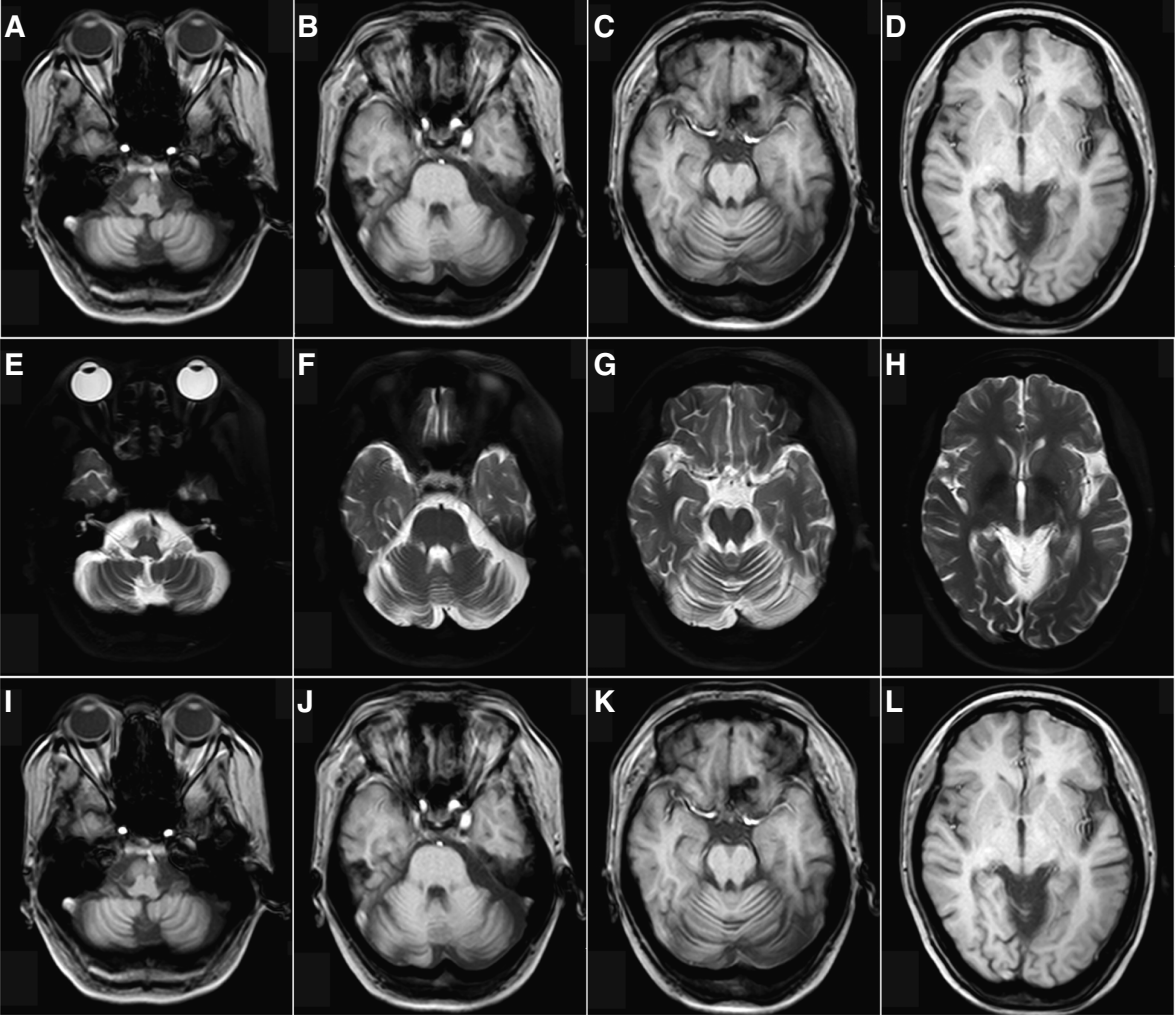
Diffuse brain and cerebellar atrophy, especially the cerebellum. Sulcus inbilateral frontal, parietal, occipital lobe increases in width and depth (**a**-**d**: T1 weighted imaging, **e**-**h**: T2 weighted imaging, **i**-**l**: Fluid attenuated inversion recovery)

Electrocardiogram investigation demonstrated sinus bradycardia (44/min on average). Nerve conduction studies revealed significant deceleration of motor conduction velocity in the right peroneal nerve (25.9 m/s), right median nerve (38.4 m/s), left ulnar nerve (45.5 m/s), right ulnar nerve (35.6 m/s) and the prolongation of F wave latency. The sensory nerve action potential could not be evoked in the bilateral peroneal nerve, sural nerve, and left median ulnar nerves. Electroencephalogram demonstrated mild to moderate abnormality and showed basic rhythms as 7.2–10 Hz and 20–40 μV slow alpha activities with low amplitude and irregular wave. Few 14–25 Hz and 20–30 μV beta waves sporadically emerged and more 4–7 Hz and 20–40 μV theta waves presented sporadically or in short-term in each lead. Visual evoked potential showed the extending of bilateral latency of P100 (Left = 101.1 mS, right = 107.0 mS) and decreased amplitude (Left = 3.24 μV, right = 1.30 μV). Brainstem auditory evoked potential showed bilateral disappearance of III-I, V-I and V-III IPL. Brain MRI revealed diffuse cerebral and cerebellar atrophy (Fig. 1).

**Fig. 2:**
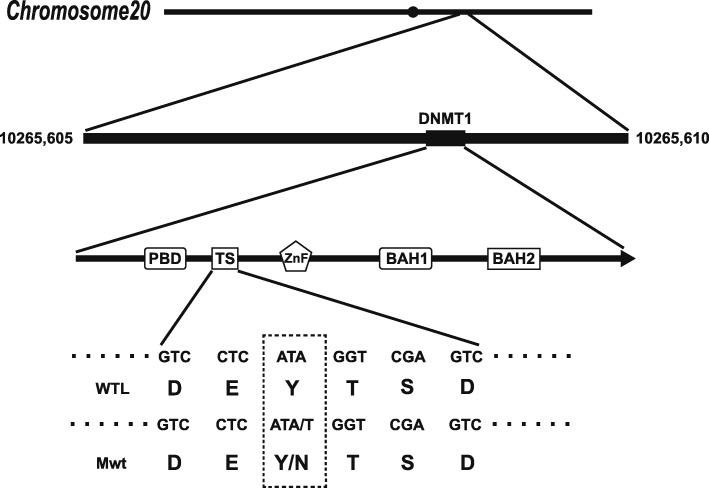
Schematic representation of DNMT1 gene structure and its multiple domains in the N-terminal region. The boxes indicate PBD (the proliferating cell nuclear antigen-binding domain), TS (the replication focus targeting sequence), ZnF (zinc finger), BAH (bromo-adjacent homologydomain).The position of the mutation in the proband is indicated in the dotted box. The sequence data for mutation was deconvoluted to indicate a novel heterozygous missense variant. The tyrosine (Y) residue at amino acid position 540 is replaced with asparagine amino acid residue.

Exome sequencing [6] (as published) performed in AmCare Genomics Lab revealed a novel heterozygous missense variant, c 1618 T > A (p. Y540N), in exon 20 of the DNMT1 (Fig. 2; Additional file 1).

## Discussion and conclusions

We report a Chinese patient with suspected HSAN1E, which was finally confirmed by exome sequencing. HSAN1E is an autosomal dominant neural degenerative disorder related to the central and peripheral nervous systems characterized by sensory loss, deafness and dementia. Our patient appeared clinically normal until developing ataxic gait at hers 30s then abnormal behavioral presentations, following by hearing loss in hers 40s, It is different from the stereotypic progressive onset of hearing loss and sensory neuropathy at first and developed mental decline later. SPECT, PET image and autopsy studies in a Japanese kinship suggested the potential frontal lobe involved and abnormal behavioral presentations [7, 8]. In contrast, a previous reported study about a large American family with 11 patients clinically examined but without behavioral presentations showed that a global process without selective frontal or subcortical involvement was indicated by brain autopsy of 3 affected patients, imaging studies and multiple neuropsychometric data [7]. It is worthy to noting that although our patient appeared with abnormal behavioral presentations and mental retardation observed by neurologist, diffuse cerebral and cerebellar atrophy instead of only frontal lobe involvement. Nerve conduction studies revealed sensory involvement. Therefore, her significant cerabellar atrophy and loss of deep sensation lead to atoxic gait. All these findings were consistent with HSAN1E but different from typical HSAN1E. Additionally, recent study further supports that the extent of the phenotypic spectrum in DNMT1-related disorders is much broader and more common than currently appreciated and estimated [9].

It is noteworthy that our patient has a novel variant c 1618 T > A (p.Y540N) in DNMT1 in exon 20. Using Sanger sequencing, we did not found the same variant in her mother. Unfortunately, we could not perform Sanger sequencing in her father since her father had already passed away at that time. However, although he did not appear any neurological manifestations until his death, we cannot make sure whether he carried the same mutation or not. Her siblings were not willing to accepting Sanger sequencing. But considering about her father and siblings without any neurological symptoms, we assume that her father should not have the variant. The identification heterozygous mutations in DNMT1 in patients have been published [2, 9]. Therefore, we determined that c 1618 T > A (p. Y540N) was a pathogenic variant. So far, the mutation, c 1618 T > A (p. Y540N), has not been discovered in our or other genetic database, neither published in clinical reports. Based on these results, we made a conclusion that the proband is likely to having a novel heterozygous pathogenic missense variant responsible for these symptoms.

In this study, we found a novel heterozygous missense variant, c 1618 T > A (p. Y540N) in exon 20 of the DNMT1, which is associated with HSAN1E. Meanwhile, both clinical assessment and genetic tests of this patient are quite different from the typical HSAN1E. Our findings expand the spectrum of DNMT1-related disorders and provide a good example of precision medicine through the combination of exome sequencing and clinical testing.

## Additional file

Additional file 1: The sequence data for novel mutation in DNMT1. The sequence data reveals a novel heterozygous missense variant, c 1618 T > A (p. Y540N), in exon 20 of the DNMT1. The tyrosine (Y) residue at amino acid position 540 is replaced with asparagine amino acid residue. (PDF 255 kb)

## Abbreviations

ADCA-DN: Autosomal dominant cerebellar ataxia, deafness and narcolepsy
BAH: Bromo-adjacent homology
DMAP1-binding domain: DNA methyltransferase-associated protein 1
DNMT1: DNA methyltransferase 1
HSAN1E: Hereditary sensory and autonomic neuropathy type IE with dementia and deafness
PBD: Proliferating cell nuclear antigen-binding domain
TS: Replication focus targeting sequence
